# Relations between specific athleticism and morphology in young basketball players

**DOI:** 10.3389/fspor.2023.1276953

**Published:** 2023-10-09

**Authors:** Nedim Čović, Denis Čaušević, Cristina Ioana Alexe, Babina Rani, Corina Ramona Dulceanu, Ensar Abazović, Gabriel Stanica Lupu, Dan Iulian Alexe

**Affiliations:** ^1^ Faculty of Sport and Physical Education, University of Sarajevo, Sarajevo, Bosnia and Herzegovina; ^2^ Department of Physical Education and Sports Performance, Faculty of Movement, Sports and Health Sciences, “Vasile Alecsandri” University of Bacau, Bacau, Romania; ^3^ Department of Physical Rehabilitation & Medicine (Physiotherapy), Post Graduate Institute of Medical Education and Research, Chandigarh, India; ^4^ Department of Physical Education and Sport Performance, Faculty of Physical Education and Sport, Aurel Vlaicu University of Arad, Arad, România; ^5^ Department of Physical and Occupational Therapy, Faculty of Movement, Sports and Health Sciences, “Vasile Alecsandri” University of Bacau, Bacau, Romania

**Keywords:** team sports, performance, fitness, body composition, power

## Abstract

**Introduction:**

Basketball is a fast-paced intermittent team sport, wherein the players must exhibit different morphologies and fitness levels depending on their position. The aim of this study was to assess the differences in the athleticism of basketball players by playing positions and in its relations with body composition and anthropometric measures. It was hypothesized that calculated athleticism has stronger and better predictive relations with morphology compared to motoric tests alone.

**Methods:**

47 basketball players were divided into three groups according to playing position [guards (n = 14), forwards (n = 22), centers (n = 11)]. Body composition and anthropometrical measurements were done for all players. Athleticism was presented in terms of overall (OFS), jumping (JFS) and sprinting (SFS) fitness scores.

**Results:**

Fitness scores were found to be more strongly related to body composition and anthropometry measurements than motoric tests alone. All three fitness scores were moderate to strongly correlated with skinfold thickness and fat percentage, while body height, fat-free mass, and lean body mass were positively correlated. Significant differences in athleticism fitness scores were found between guards and other groups. Forwards displayed superior athleticism in power and sprint abilities when compared to guards and centers. Multivariate logistic regression revealed that fat percentage, lean muscle mass, skinfold thickness, and arm span exhibited strong predictive capabilities in relation to athleticism scores.

**Conclusions:**

Coaches and practitioners should be aware that athleticism includes a multitude of components, and they should use athleticism assessments before designing training regimens that are tailored to each position's unique needs.

## 1. Introduction

Physical activity in basketball is composed of intense intermittent running, short sprinting, and power-jumping tasks (1, 2). Basketball players showed different physical and physiological features, which is especially noticed when compared between playing positions (3–6).

As a fast-paced team sport, basketball involves a series of dynamic movements in a variety of directions (7). During the game, the body decelerates and reaccelerates with each change of movement or direction, using highly precise force transmission (8). Therefore, a basketball player's performance on the court mainly depends on speed and power-related attributes differentiated by playing levels (7) and playing positions (9). Guards typically perform better in power scoring than centers (9–11). Inevitably, the guards are expected to perform best during running, single-leg jumping, and agility *T*-test, while the high quality of short-distance speed, as well as lower and upper body strength, are expected from all the players (9, 12). However, few studies have depicted no significant differences between positions in explosive power during vertical jump performance (13, 14). Since the on-court physical demands vary for the playing positional role, therefore the training of players must be individualized considering the positional variations, with the position-specific physical drills design (15).

Anthropometric characteristics such as body height, skinfold thickness, body circumference, and fat percentage are often used to discern a player's level of performance (16). Elite-level centers are heavier and taller than guards and forwards along with higher fat mass than forwards, and higher fat-free mass than guards (10, 17). Thus, it is important to consider body height when assigning specific positions to the players (18). Given playing position, guards tend to have dominantly endomorphic somatotype features relative to forwards (10) while a study by Gryko et al. (17) has revealed that young players are more ectomorphic compared to adult professional players that are predominantly mesomorph types. Therefore, profiling the physical and physiological attributes of basketball players must be considered with respect to age, playing level, and position. This is especially important when attempting to customize the training program or for player selection. The aim must be the balanced development of a player, focusing on speed, agility, power, coordination, specific skill set, tactics, techniques, and psychology, in association with the dimensional profile support, growth, and maturation of young athletes.

The anthropometric profile in senior-level basketball players has been demonstrated to be directly connected to multiple motor performance variables (19–21). In the case of basketball, successful performance depends on a player's jumping ability simultaneously with the shots or rebounds (22). A player's vertical jumping performance directly impacts his level of play, as the former is an integral part of different game moves like rebounds, layup shots, basket pitches, dunks, and rebounds. Categorically, the height and arm span correlated well with the one-hand-shot block and rebound at two hands, but weakly with the squat jump and countermovement jump (19). Another study by Ribeiro et al. (21) reported a strong correlation of relative power with fat mass and flight time, concluding that players with higher lean body mass and lower fat percentage depicted superior jumping performance. These findings were supported by Nunes et al. (20) in their study on Brazilian women's basketball players. The body fat percentage greater than the population mean (8%–22%) (23) can prove to be a hindering block for young male basketball players. Consequently, the correct identification of the player's morphology and anthropometry is crucial to the sport's performance, owing to its close linkage with the player's technical role as well as for the specific jumping and sprinting skills.

Specific athleticism is assessed using a variety of speed and power fitness tests (24). Considering current knowledge about the specific fitness tests (speed, power, agility) to the basketball players' performance, it becomes difficult to project the results to a particular factor as more or less determining. As such, it might be beneficial to get a clear and concise picture with a panoramic view of physical fitness tests. Therefore, to provide a holistic glimpse of the player's fitness with a single score, the concept of Total Score of Athleticism (TSA) emerged (25). TSA is derived from the sum of *Z*-scores obtained using data from a fitness testing battery, based on the top (red) and bottom (green) thirds of all scores. TSA is further divided into red, amber, and green zones (25). Moreover, critical issues of differences in fitness between playing position and their relations with morphology are compromised by different testing protocols, test inconsistencies, lack of longitudinal studies and small sample size used (26). Rather than analyzing individual test results separately, creating a composite score that integrates various performance characteristics offers a more comprehensive perspective, explaining a greater amount of variance (27). The use of TSA, comprising a series of tests, enables coaches to assess athletes within the context of their team or peers with similar training regimens. This facilitates the establishment of realistic benchmarks and training goals tailored to the club's specific demands (28).

Comparative data from other teams may reflect specific fitness characteristics associated with a particular style of play, which may not be universally applicable. Consequently, for talent identification purposes, comparing athletes to one another is the most advantageous application of TSA. TSA and *Z*-scores, due to their unit-less nature, enable comparisons across various tests that would otherwise be impractical. In talent identification, *Z*-scores can be effectively employed from a young age, as they indicate how many standard deviations an individual deviates from the mean (25, 28). Roughly, around 4% of athletes fall within ±3 standard deviations, with 2% classified as high performers (+3SD). In some extreme instances, exceptionally talented individuals may achieve test scores (or TSA scores) exceeding +4 SD. Prediction models using anthropometric and fitness characteristics can predict future performance in adulthood with a reasonable degree of precision (29). Nonetheless, considering talent identification it is crucial to consider various factors including maturity status, biological age, training age, training allocation, testing procedures, and the status of cohorts and athletes (recreational, semi-professional, or professional) (25, 28, 30).

Recent studies have shown that TSA (30) or Composite Scores of Readiness (CSRs), as named in Oleksy et al. (31), derived from isokinetic strength assessments, jumps (TSA study), and Functional Movement Screen, *Y*-Balance Test, and Tuck Jump Assessment (CSR study), provide better insights into performance levels following ACL injuries in soccer players. They also offer greater differentiation between individuals with significant functional deficits and those without injuries or with minor deficits.

Identifying the factors that determine training load and athletic performance is one of the tasks undertaken by sports scientists or team trainers (32). With athleticism as an under-researched parameter, it becomes imperative to explore its relationship with the morphology, playing position, and sport performance of basketball players. Hence, the present study aimed to assess the differences in the athleticism of basketball players by playing positions and in its relations with body composition and anthropometric measures. We hypothesized that calculated athleticism has stronger and better predictive relations with morphology compared to motoric tests alone.

## 2. Materials and methods

### 2.1. Participants

Sample calculation was performed via online available G*Power software (ver. 3.1.9.7) (33) using Multivariate linear regression was used as type of statistic, effect size was set at medium (0.5), statistical significance at *α* = 0.05, research power at 0.80 and the number of predictors 13 (all included variables). Calculation determined total sample size no less than *n* = 41. Inclusion criteria mandated that all participants possess a minimum of four years of prior playing experience, while the exclusion criteria encompassed the following conditions: (i) the occurrence of a severe injury within the preceding six months, (ii) an increase in peak height exceeding 7 cm within the last 12 months to avoid sample heterogeneity (34), and (iii) demonstrated inability to undertake motor tests. In total, 47 basketball players from five top-ranking teams of BiH national League volunteered to participate in the study. All players had on average 4.6 ± 0.2 years of training experience, have had recent national-level competition experience within the last two years and have been actively involved in a weekly training schedule that includes five 120-min basketball practice sessions and one game. They were distributed in 3 groups according to playing position: (a) Guards [*n* = 14; age (mean ± SD): 14.29 ± 0.61 years.; height: 173.56 ± 5.55 cm and body mass: 59.96 ± 9.91 kg] encompassed positions 1 and 2 (PG—point guard and SG—shooting guard), (b) Forwards [*n* = 22; age (mean ± SD): 15.26 ± 0.82 years.; height: 184.31 ± 4.06 cm and body mass: 70.36 ± 7.29 kg] encompassed positions 3 and 4 (SF—shooting forward and PF—power forward), and (c) Centers [*n* = 11; age (mean ± SD): 15.33 ± 0.63 years.; height: 191.05 ± 6.32 cm and body mass: 85.15 ± 9.70 kg] which only included position 5 (C - centers). Procedures did not utilize nor calculate maturity status, which can significantly impact performance. This omission led to the utilization of exclusion criterion ii). The findings regarding body height and weight indicate that guards exhibited a lesser degree of development in comparison to forwards and centers. All subjects (including parents and coaches) were informed in detail of the aim and experimental procedures of the study and signed informed consent for participation.

### 2.2. Procedures

Testing procedures were performed at Institute of Sport, Faculty of Sport and Physical Education, University of Sarajevo. The testing session lasting two days (in a row) was conducted prior to pre-competitive mesocycle in the morning between 10:00 and 13:00 h. For the first day, players were asked to refrain from any intense exercise or activity and eating for 2 h before testing. On the first day, testing procedure included only body composition and anthropometry measurements. The following day, all sprint and jumping tests were performed. Prior to the testing procedure, all players performed standardized warm-up protocol (3, 35). All the tests and measurements were performed by trained personnel according to standardized procedures. The study was approved by the Ethics Committee (No: 01-2603/22; 7 January 2022) of the Faculty of Sports and Physical Education, University of Sarajevo, and was carried out in accordance with the Declaration of Helsinki. Informed consent was obtained from the participants' parents or legal guardians, prior to enrolment into the study.

#### 2.2.1. Body composition and anthropometry

Trained anthropometrics measured height, weight, skinfolds (four sites), arm span, hand span and standing reach using standard protocol for all subjects. For each test procedure, the same examiner measured all individuals. Standing height was measured using a digital stadiometer (InBody BSM 370; Biospace Co., Ltd., Seoul, Republic of Korea). Body weight, fat free mass (FFM), fat percentage (BF%), lean body mass (LBM%), body mass index (BMI) was estimated using a direct segmental high-frequency bioelectrical impedance scale (InBody 720; Biospace Co., Ltd., Seoul, Republic of Korea). All subjects were barefoot and wearing only underwear during the test. Skinfold thickness (suprailiac, subscapular, biceps, triceps) was measured using a caliper (SATA, Seville, Spain). Arm span and hand span were measured (in cm) with a measuring tape (SATA, Seville, Spain). Standing reach was measured with 0.1 cm accuracy while the participant was standing comfortably using the Vertec Vertical Jump Trainer (Sports Imports, Hilliard, OH, USA). BMI was calculated as a fraction of body weight and a square of the standing height (kg/m2).

#### 2.2.2. Sprint tests

From a standing position, a 20 m sprint was performed with the front foot 20 cm behind the starting line to prevent premature time start. Sprint time was recorded by five infrared timing gates (1.2 m height and 1.5 m wide) (Photocells; Microgate, Bolzano, Italy), placed on starting line, at 5, 10, 15 and 20 m. All players were allowed two trials, with a 3-minute rest between, and the best time was used as result and for statistical analysis.

#### 2.2.3. Jump tests

The following five types of jumps were performed: countermovement jump (CMJ), countermovement jump free arms (CMJ free arms), drop jump from 40 cm (DJ40), Stiffness 10 jumps (STF10), standing long jump (SLJ). All protocols included two trials and were measured by using (except SLJ) OptoJump Next system (Microgate, Bolzo, Italy). CMJ, CMJ free arms and DJ40 tests were carried out in accordance with recognized protocols (35, 36). In order to prevent any arm effect, hands were kept at the hips during the whole movement in CMJ and DJ40. Drop jump was performed from 40 cm wooden box, as recommended by previous study (37). For STF10, subjects performed 10 consecutive maximal jumps, while keeping their legs as straight as possible and their hands on the hips (38). The performance was determined from these jumps in terms of average jump height.

The SLJ test required the subjects to perform a maximal horizontal jump from a standing still position. The overall jump distance, which is the horizontal distance from the take-off line to the mark made by the heel on landing, was measured in cm. No backward steps or forward hops/runs were permitted.

### 2.3. Overall, jumping and sprinting athleticism fitness score

Specifically for this study, athleticism was quantified using test results and presented as Overall Fitness Score (OFS), Jumping Fitness Score (JFS) and Sprinting Fitness Score (SFS) like the TSA calculation (25). Athleticism scores based on *Z*-scores provide practitioners with a means to compare data among homogenous athletes characterized by congruent training regimens, requisites, and limitations. Consequently, test scores are presumed attainable by all athletes creating pragmatic benchmarks and goals towards development can be directed. The weighting assigned to motor ability tests also affects athleticism scores. Athleticism scores may be skewed toward a particular ability over others within certain assessments involving composite measures that assess various motor abilities. When calculating athleticism scores, it is mandatory to select tests judiciously, considering the expertise of practitioners and the predominant skills required for specific playing positions. Present study tests are used effectively to mitigate the potential bias related to other than power and running velocity capabilities.

Results for jumps (CMJ, CMJ free arms, DJ 40 cm, STF10 jumps, standing long jump) and sprints (5 m, 10 m, 15 m and 20 m) were converted to Z – scores. To compute the Z-scores for each test, the formula employed was as follows: *Z*-score = (Player's score - Mean of the Cohort)/Standard Deviation of the Cohort (30). OFS, JFS and SFS presented sum of *Z*-scores for jumps and inverted *Z*-scores for sprinting standardized by the number of tests used (9 for OFS, 5 for JFS and 4 for SFS) to avoid the influence of different numbers of tests used.

### 2.4. Statistical analysis

The Kolmogorov-Smirnov test was performed to check normality of data distribution while data homogeneity was checked using Levene's test. Two-way ANOVA and Bonferroni *post hoc* tests were performed for comparisons between groups by playing position. Pearson's correlation coefficient tests were performed to identify the relationships between age, morphology, overall, jumping, and sprinting fitness scores. Correlations were determined as follows: *r* < 0.19 as very weak, 0.2–0.39 as weak, 0.40–0.59 as moderate, 0.6–0.79 as strong and 0.8–1.0 as very strong (39). Additionally, to determine the variety of the correlation differences between playing positions for fitness scores, limits for decision of the differences for Pearson *r* correlations were calculated using an online available Excel spreadsheet (40). Furthermore, predictive relationship of athleticism fitness scores and morphology was performed using stepwise multivariate linear regression analysis. Effect sizes (ES) of the differences between fitness scores were calculated and interpreted based on Cohen's d thresholds of >0.2 - small, >0.5 – moderate, >0.8 – large and >1.3 very large (41). SPSS software (Ver.21, Chicago, IBM) was used for data analysis. Statistical significance was set at *p* < 0.05. Bonferroni correction was used to adjust the *p*-value for the comparison difference for OFS, JFS and SFS between playing positions. Considering the research's sampling methodology, a *post hoc* power analysis conducted utilizing G*Power (Version 3.1.9.7) unveiled that the statistical power for within-group comparisons was as follows: 91.7% for the detection of a large effect size, 55.0% for a moderate effect size, and 10.6% for a small effect size.

## 3. Results

The Levene's and Kolmogorov – Smirnov tests indicated that homogeneity and normality of data distribution were adequate for all tests. Significant moderate to strong correlations (*p* < 0.001) were present between jump tests (CMJ, CMJ free arms, DJ 40 cm, STF10 jumps and SLJ) and sprint tests (5 m, 10 m, 15 m and 20 m) (Table 1). Strong to very strong correlations (*p* < 0.001) were noted between OFS, JFS and SFS (Table 2).

**Table 1:** Pearson's *r* correlations between jump (CMJ, CMJ free arms, DJ 40 cm, STIFFNESS 10 jumps and standing long jump) and sprint tests (5 m, 10 m, 15 m, and 20 m) for young basketball players (*n* = 47).

	5 m	10 m	15 m	20 m
CMJ	−.550**	−.291*	−.725**	−.763**
CMJ free arms	−.500**	−.269	−.613**	−.701**
DJ 40 cm	−.423**	−.257	−.678**	−.715**
STIFFNESS 10 jumps	−.476**	−.227	−.654**	−.667**
Standing long jump	−.442**	−.321*	−.661**	−.707**

CMJ, Countermovement jump.

DJ 40 cm, Drop jump from 40 cm.

* Correlation is significant at the 0.05 level (2-tailed).

** Correlation is significant at the 0.001 level (2-tailed).

**Table 2:** Pearson's *r* intercorrelations between overall (OFS), jumping (JFS) and sprinting (SFS) athleticism fitness scores for young basketball players (*n* = 47).

	OFS	JFS	SFS
Overall Fitness Score (OFS)	1	.937**	.894**
Jumping Fitness Score (JFS)	.937**	1	.682**
Sprinting Fitness Score (SFS)	.894**	.682**	1

** Correlation is significant at the 0.001 level (2-tailed).

Table 3 displays differences between playing positions for all the body composition, anthropometry, and motor tests as well as for athleticism fitness scores. Guards were significantly younger compared to forwards and centers (mean ± SD: 0.97 ± 0.71 and 1.04 ± 0.70 years.) (Table 3), but non-significant relation of age was noted for the set of jump and sprint tests as well as OFS, JFS and SPS. When compared to forwards and centers, guards had significantly lower OFS by MD = −0.82 (ES = −1.22, large) and MD = −0.70 (ES = −0.95, large), JFS by MD = −0.40 (ES = −1.12, large) and MD = −0.37 (ES = −0.79, moderate), and SFS by MD = −0.43 (ES = −1.02, large) and MD = −0.33 (ES = −0.82, large) (Supplementary Figure S1).

**Table 3:** Body composition, anthropometric measures and athleticism fitness scores of young basketball players.

	Overall (*n* = 47)	Guards (*n* = 14)	Forwards (*n* = 22)	Centers (*n* = 11)		
	Mean ± SD	Mean ± SD	Mean ± SD	Mean ± SD	*F*	*p*
Overall Fitness Score (OFS)	0.00 ± 0.80	−0.55 ± 0.64^*,#^	0.27 ± 0.70	0.15 ± 0.82	5.698	0.006
Jumping Fitness Score (JFS)	0.00 ± 0.89	−0.55 ± 0.68^*,#^	0.25 ± 0.75	0.19 ± 1.14	4.297	0.020
Sprinting Fitness Score (SFS)	0.00 ± 0.87	−0.56 ± 0.89^*,#^	0.30 ± 0.79	0.10 ± 0.72	5.010	0.011
Age	14.99 ± 0.84	14.29 ± 0.61^*,#^	15.26 ± 0.82	15.33 ± 0.63	9.247	<0.001
Body height (cm)	182.69 ± 8.26	173.56 ± 5.55^*,#^	184.31 ± 4.06^#^	191.05 ± 6.32	38.26	<0.001
Body mass (kg)	70.72 ± 12.55	59.96 ± 9.91^*,#^	70.36 ± 7.29^#^	85.15 ± 9.70	25.85	<0.001
BMI (kg/m^2^)	21.05 ± 2.57	19.84 ± 2.75^#^	20.71 ± 1.92^#^	23.29 ± 2.23	7.664	0.001
Fat free mass (%)	62.73 ± 10.66	52.84 ± 7.94^*,#^	63.10 ± 6.09^#^	74.59 ± 8.39	27.92	<0.001
Fat % (%)	7.99 ± 3.79	7.12 ± 3.31^#^	7.25 ± 3.32^#^	10.56 ± 4.37	3.708	0.033
Lean body mass (%)	35.35 ± 6.47	29.32 ± 4.92^*,#^	35.64 ± 3.76^#^	42.45 ± 5.01	27.19	<0.001
Standing reach (cm)	238.05 ± 10.96	226.82 ± 7.25^*,#^	239.85 ± 6.60^#^	248.75 ± 9.13	28.03	<0.001
Arm span (cm)	186.37 ± 10.00	176.41 ± 7.17^*,#^	188.20 ± 6.21^#^	195.38 ± 8.75	23.09	<0.001
Hand span (cm)	22.54 ± 1.52	21.50 ± 1.36^*,#^	22.73 ± 1.20	23.48 ± 1.62	7.038	0.002
Suprailiac skinfold (mm)	16.66 ± 9.36	12.79 ± 5.99^#^	15.25 ± 7.44^#^	24.41 ± 12.19	6.464	0.003
Subscapular skinfold (mm)	11.13 ± 3.88	9.68 ± 2.91^#^	10.70 ± 3.66^#^	13.82 ± 4.35	4.279	0.020
Biceps skinfold (mm)	11.83 ± 4.20	10.36 ± 2.20^#^	11.52 ± 4.21	14.32 ± 5.27	3.106	0.055
Triceps skinfold (mm)	6.25 ± 2.32	5.82 ± 1.87	5.89 ± 1.98	7.55 ± 3.09	2.365	0.106
CMJ (cm)	31.87 ± 5.29	28.56 ± 4.08^*,#^	33.38 ± 4.61	33.07 ± 6.35	4.514	0.016
CMJ free arms (cm)	40.25 ± 6.47	37.06 ± 6.32*	41.88 ± 4.98	41.04 ± 8.24	2.651	0.082
DJ 40 cm (cm)	31.40 ± 5.02	28.62 ± 3.39*	32.79 ± 4.44	32.16 ± 6.62	3.452	0.041
STIFFNESS 10 jumps (cm)	29.02 ± 4.95	27.13 ± 4.17^#^	29.20 ± 5.07	31.06 ± 5.15	2.062	0.139
Standing long jump (cm)	218.02 ± 26.01	200.14 ± 16.66^*,#^	228.55 ± 23.56	219.73 ± 29.87	6.317	0.004
5 m (s)	1.13 ± 0.10	1.18 ± 0.12	1.10 ± 0.09^¥^	1.13 ± 0.08	2.467	0.096
10 m (s)	1.95 ± 0.20	2.06 ± 0.26	1.87 ± 0.13^¥^	1.95 ± 0.17	4.467	0.017
15 m (s)	2.56 ± 0.18	2.66 ± 0.19	2.51 ± 0.17^¥^	2.52 ± 0.15^¥^	3.572	0.037
20 m (s)	3.24 ± 0.21	3.38 ± 0.22	3.18 ± 0.20^¥^	3.20 ± 0.17^¥^	4.579	0.016

*p*, significance of the differences between Guards, Forwards and Centers; *F*, *F* ratio; BMI, body mass index; CMJ, countermovement jump; DJ, drop jump.

^#^ Significantly lower at *p* < 0.05 to Centers.

* Significantly lower at *p* < 0.05 to Forwards.

^¥^ Significantly lower at *p* < 0.05 to Guards.

Moderate to strong significant (*p* < 0.05) correlations (Table 4) were observed between OFS and body height (*r* = 0.53), biceps (*r* = −0.63) and triceps (*r* = −0.71) skinfolds; JFS and body height (*r* = 0.62), fat free (*r* = 0.63) and lean body mass (*r* = 0.64); as well as between SFS and fat percentage (*r* = −0.69) along with all skinfold's thickness (*r* = −0.71 to −0.58) in guards.

**Table 4:** Correlations between overall fitness score (OFS), jumping fitness score (JFS) and sprinting fitness score (SFS) and body morphology measures in young basketball players.

	Overall; (*n* = 47)			Guards (*n* = 14)			Forwards (*n* = 22)			Centers (*n* = 11)		
	OFS (*r*) 95%CI [−,+]	JFS (*r*) 95%CI [−,+]	SFS (*r*) 95%CI [−,+]	OFS (*r*) 95%CI [−,+]	JFS (*r*) 95%CI [−, +]	SFS (*r*) 95%CI [−,+]	OFS (*r*) 95%CI [−,+]	JFS (*r*) 95%CI [−,+]	SFS (*r*) 95%CI [−,+]	OFS (*r*) 95%CI [−, +]	JFS (*r*) 95%CI [−,+]	SFS (*r*) 95%CI [−,+]
Body height	.421** **[0.15, 0.63]**	.418** **[0.14, 0.63]**	.348* **[0.07, 0.58]**	0.527* **[−0.00, 0.83]**	.621* **[0.13, 0.87]**	0.3 [−0.27, 0.72]	−0.2 [−0.57, 0.24]	−0.24 [−0.6, 0.21]	−0.13 [−0.52, 0.31]	0.41 [−0.25, 0.81]	0.41 [−0.25, 0.81]	0.3 [−0.37, 0.76]
Body mass	0.22 [−0.07, 0.48]	0.28 [−0.01, 0.52]	0.1 [−0.19, 0.38]	0.09 [−0.46, 0.59]	0.45 [−0.1, 0.79]	−0.28 [−0.7, 0.3]	−0.06 [−0.47, 0.37]	0 [−0.42, 0.42]	−0.12 [−0.52, 0.32]	−0.05 [−0.63, 0.57]	−0.07 [−0.64, 0.55]	0.02 [−0.59, 0.61]
BMI	0.02 [−0.27, 0.3]	0.12 [−0.18, 0.39]	−0.11 [−0.39, 0.18]	−0.14 [−0.63, 0.42]	0.25 [−0.33, 0.69]	−0.48 [−0.81, 0.07]	0.01 [−0.43, 0.41]	0.1 [−0.34, 0.5]	−0.09 [−0.49, 0.34]	−0.33 [−0.77, 0.34]	−0.36 [−0.79, 0.31]	−0.17 [−0.7, 0.48]
Fat free mass	.406** **[0.13, 0.62]**	.434** **[0.17, 0.64]**	.295* **[0.01, 0.54]**	0.32 [−0.26, 0.73]	.624* **[0.14, 0.87]**	−0.06 [−0.57, 0.49]	0.24 [−0.21, 0.6]	0.23 [−0.21, 0.59]	0.21 [−0.23, 0.58]	0.27 [−0.39, 0.75]	0.22 [−0.44, 0.72]	0.3 [−0.37, 0.76]
Fat %	−.417** **[−0.63, −0.15]**	−.301* **[−0.54, −0.02]**	−.487** **[−0.68, −0.23]**	−0.48 [−0.81, 0.07]	−0.14 [−0.62, 0.42]	−.688** **[−0.89, −0.25]**	−.561** **[−0.79, −0.18]**	−0.42 [−0.72, −0.00]	−.648** **[−0.84, −0.31]**	−.624* **[−0.89, −0.04]**	−0.58 [−0.88, 0.03]	−0.53 [−0.86, 0.1]
Lean body mass	.422** **[0.15, 0.63]**	.450** **[0.19, 0.65]**	.309* **[0.024, 0.55]**	0.34 [−0.24, 0.74]	.640* **[0.17, 0.87]**	−0.04 [−0.56, 0.5]	0.26 [−0.18, 0.62]	0.26 [−0.18, 0.61]	0.23 [−0.21, 0.6]	0.29 [−0.38, 0.76]	0.24 [−0.42, 0.73]	0.32 [−0.35, 0.77]
Standing reach	.363* **[0.09, 0.59]**	.385** **[0.09, 0.59]**	0.27 [−0.02, 0.51]	0.3 [−0.27, 0.72]	0.4 [−0.17, 0.77]	0.13 [−0.43, 0.62]	−0.24 [−0.6, 0.2]	−0.26 [−0.61, 0.19]	−0.19 [−0.57, 0.25]	0.48 [−0.17, 0.84]	0.53 [−0.1, 0.86]	0.25 [−0.41, 0.74]
Arm span	.402** **[0.13, 0.62]**	.434** **[0.17, 0.64]**	0.29 [−0.00, 0.53]	0.31 [−0.27, 0.72]	0.46 [−0.09, 0.8]	0.08 [−0.47, 0.59]	−0.05 [−0.46, 0.38]	−0.08 [−0.48, 0.36]	−0.02 [−0.43, 0.41]	0.43 [−0.23, 0.82]	0.51 [−0.13, 0.85]	0.16 [−0.49, 0.69]
Hand span	.352* **[0.07, 0.58]**	.305* **[0.02, 0.54]**	.347* **[0.06, 0.58]**	0.04 [−0.5, 0.56]	0.1 [−0.45, 0.6]	−0.02 [−0.55, 0.51]	0.04 [−0.39, 0.45]	−0.08 [−0.49, 0.35]	0.18 [−0.26, 0.56]	0.57 [−0.05, 0.87]	0.45 [−0.21, 0.82]	.651* **[0.08, 0.9**]
Suprailiac skinfold	−.306* **[−0.55, −0.021]**	−0.23 [−0.49, 0.06]	−.344* **[−0.57, −0.06]**	−0.38 [−0.76, 0.18]	−0.04 [−0.56, 0.5]	−.614* **[−0.86, −0.12**]	−0.4 [−0.7, 0.03]	−0.35 [−0.67, 0.08]	−0.4 [−0.7, 0.03]	−.725* **[−0.92, −0.22]**	−.605* **[−0.88, −0.01]**	−.761** **[−0.93, −0.3]**
Subscapular skinfold	−0.24 [−0.49, 0.05]	−0.18 [−0.45, 0.11]	−0.27 [−0.52, 0.021]	−0.28 [−0.29, 0.71]	0.1 [−0.45, 0.6]	−.582* **[−0.85, −0.07]**	−0.23 [−0.59, 0.21]	−0.25 [−0.61, 0.19]	−0.18 [−0.56, 0.26]	−.740** **[−0.93, −0.25]**	−.641* **[−0.9, −0.07]**	−.729* **[−0.92, −0.23]**
Biceps skinfold	−.326* **[−0.56, −0.043]**	−0.28 [−0.53, 0.00]	−.323* **[−0.56, −0.04]**	−.625* **[−0.87, −0.14]**	−0.39 [−0.76, 0.17]	−.690** **[−0.89, −0.25]**	−.500* **[−0.76, −0.01]**	−.427* **[−0.72, −0.00]**	−.518* **[−0.77, −0.12]**	−0.47 [−0.83, 0.19]	−0.46 [−0.83, 0.19]	−0.34 [−0.78, 0.32]
Triceps skinfold	−.501** **[−0.69, −0.25]**	−.422** **[−0.63, −0.15]**	−.509** **[−0.69, −0.26]**	−.712** **[−0.9, −0.29]**	−0.53 [−0.83, 0.00]	−.711** **[−0.9, −0.29]**	−.531* **[−0.78, −0.14]**	−0.42 [−0.71, 0.00]	−.595** **[−0.81, −0.23]**	−.722* **[−0.92, −0.22]**	−.647* **[−0.9, −0.08]**	−.668* **[−0.91, −0.11]**

*r*, Pearson correlation coefficient; 95%CI [-,+], 95% Confidence interval [Lower, Upper]; OFS, Overall Fitness Score;

JFS, Jumping Fitness Score; SFS, Sprinting Fitness Score.

* Significant at *p* < 0.05.

** Significant at *p* < 0.001.

Among forwards, significant correlations (*p* < 0.05) (Table 4) were noted for OFS and fat percentage (*r* = −0.56), biceps (*r* = −0.50) and triceps (*r* = −0.53) skinfolds; JFS and biceps skinfold (*r* = −0.43); and SFS and fat percentage (*r* = −0.65), biceps (*r* = −0.52) and triceps (*r* = −0.60) skinfolds.

In centers, OFS was significantly (*p* < 0.05) (Table 2) correlated with fat percentage (*r* = −0.62), suprailiac (*r* = −0.73), subscapular (*r* = −0.74) and triceps (*r* = −0.72) skinfolds; JFS and suprailiac (*r* = −0.61), subscapular (*r* = −0.64) and triceps (*r* = −0.65) skinfolds; as well as SFS and hand span (*r* = 0.65), suprailiac (*r* = −0.76), subscapular (*r* = −0.73) and triceps (*r* = −0.67) skinfold thicknesses. Limits of decision for the difference based on Pearson's correlations between playing positions for OFS, JFS and SFS are presented in Supplementary Table S1.

A stepwise multiple regressions (Table 5) of athleticism fitness scores revealed highly significant models for OFS: For guards (*R*^2 ^= 0.51; *p* < 0.001) with triceps skinfold thickness included as significant predictor (*β* = −0.71; *p* = 0.04), for forwards (*R*^2 ^= 0.46; *p* < 0.001) with fat percentage (*β* = −0.84; *p* = 0.001) and BMI (*β* = 0.49; *p* = 0.026) and for centers (*R*^2 ^= 0.55; *p* < 0.001) with subscapular skinfold (*β* = −0.74; *p* = 0.009), respectively.

**Table 5:** Multivariate regression models for Overall Fitness Score (OFS), Jumping Fitness Score (JFS) and Sprinting Fitness Score (SFS) with partial predictive values of body morphology measures in basketball players.

Overall Fitness Score (OFS) regression models					
Guards (*n* = 14): R = 0.712, *R^2^*= 0.507, SEE = 4.423, *F*_(1,12)_ = 12.348**					
	Standardized *β*	Unstandardized *β*	*SE*	*p*	95% CI for *β* [−; +]
Triceps skinfold	−0.712	−2.306	0.656	0.04	[−3.736; −0.876]
Forwards (*n* = 22): *R* = 0.689, *R*^2^= 0.475, SEE = 4.927, *F*_(2,19)_ = 8.586**					
	Standardized *β*	Unstandardized β	*SE*	*p*	95% CI for *β* [−; +]
Fat%	−0.836	−1.627	0.393	0.001	[−2.448; −0.805]
Body mass index	0.485	1.635	0.68	0.026	[0.212; 3.057]
Centers (*n* = 11): *R* = 0.74, *R^2^*= 0.548, SEE = 5.501, *F*_(1,9)_ = 10.896**					
	Standardized β	Unstandardized β	*SE*	*p*	95% CI for *β* [−; +]
Subscapular skinfold	−0.74	−1.321	0.4	0.009	[−2.226; −0.416]
Jumping Fitness Score (JFS) regression models					
Guards (*n* = 14): *R* = 0.82, *R^2^*= 0.673, SEE = 2.11, *F*_(2,11)_ = 11.304**					
	Standardized *β*	Unstandardized *β*	*SE*	*p*	95% CI for *β* [−; +]
Lean body mass	1.409	0.975	0.215	0.001	[0.502; 1.449]
Body mass index	−0.924	−1.145	0.385	0.013	[−1.993; −0.297]
Forwards (*n* = 22): *R* = 0.598, *R^2^*= 0.357, SEE = 3.15, *F*_(2,19)_ = 5.281**					
	Standardized *β*	Unstandardized β	*SE*	*p*	95% CI for β [−; +]
Body mass index	0.511	0.998	0.439	0.035	[0.08; 1.916]
Biceps skinfold	−0.721	−0.642	0.2	0.005	[−1.061; −0.223]
Centers (*n* = 11): *R* = 0.647, *R^2^*= 0.419, SEE = 4.58, *F*_(1,9)_ = 6.486**					
	Standardized *β*	Unstandardized *β*	*SE*	*p*	95% CI for *β* [−; +]
Triceps skinfold	−0.647	−1.195	0.469	0.031	[−2.256; −0.133]
Sprinting Fitness Score (SFS) regression models					
Guards (*n* = 14): *R* = 0.821, *R^2^*= 0.643, SEE = 2.2, *F*_(2,11)_ = 11.374**					
	Standardized β	Unstandardized *β*	*SE*	*p*	95% CI for *β* [−; +]
Triceps skinfold	−0.503	−0.955	0.366	0.024	[−1.761; −0.148]
Fat%	−0.461	−0.494	0.207	0.036	[−0.949; −0.038]
Forwards(*n* = 22): *R* = 0.729, *R^2^*= 0.532, SEE = 2.27, *F*_(2,19)_ = 10.794**					
	Standardized β	Unstandardized β	*SE*	*p*	95% CI for β [−; +]
Fat%	−0.878	−0.834	0.181	0.001	[−1.213; −0.455]
Body mass index	0.407	0.669	0.313	0.046	[0.013; 1.324]
Centers (*n* = 11): *R* = 0.761, *R^2^*= 0.0578, SEE = 1.96, *F*_(1,9)_ = 12.374**					
	Standardized *β*	Unstandardized *β*	*SE*	*p*	95% CI for β [−; +]
Suprailiac skinfold	−0.761	−0.179	0.051	0.007	[−0.294; −0.064]

*R*, Regression model correlation coefficient; *R*^2^, Regression model coefficient of determination; SEE, Standard error of estimate; SE, standard error; *p*, significance level; 95% CI for *β* [-; +], 95% confidence interval for standardized *β* [Lower; Upper].

** Significant at the 0.001 level.

Regression of JFS revealed models: For guards (*R*^2 ^= 0.67; *p* < 0.001) with lean body mass (*β* = 1.41; *p* = 0.001) and BMI (*β* = −0.92; *p* = 0.013), for forwards (*R*^2 ^= 0.36; *p* < 0.001) with BMI (*β* = 0.51; *p* = 0.035) and biceps skinfold (*β* = −0.72; *p* = 0.005) and for centers (*R*^2 ^= 0.42; *p* < 0.001) with triceps skinfold (*β* = −0.65; *p* = 0.031), respectively.

Regression of SFS revealed models: For guards (*R*^2 ^= 0.64; *p* < 0.001) with triceps skinfold (*β* = −0.50; *p* = 0.024) and fat percentage (*β* = −0.46; *p* = 0.036), for forwards (*R*^2 ^= 0.53; *p* < 0.001) with fat percentage (*β* = −0.88; *p* = 0.001) and BMI (*β* = 0.41; *p* = 0.046) and centers (*R*^2 ^= 0.58; *p* < 0.001) with suprailiac skinfold thickness (*β* = −0.76; *p* = 0.007), respectively.

## 4. Discussion

In all three groups of playing position, fitness scores were more strongly related to body composition and anthropometry measurements than motor tests alone. All three fitness scores were moderate to strongly correlated with skinfold thickness and fat percentage, while body height, fat-free mass, and lean body mass were positively correlated. A moderate to very large difference was observed between guards compared to forwards and centers in terms of athleticism fitness scores. BMI was also assumed to be a significant predictor of fitness scores based on predictive regression models. Results obtained could allow practitioners to discriminate specific skills and anthropometric features according to playing position. Complex morphological differences among playing positions appear to be the results of different growth rates, geographical and ethnic variety, and presumptions for retaining extremely high players (42, 43) thereby forgetting about the motoric potential of players as a key success factor.

As observed in previous studies (4–6, 44) present study results agree with findings among male basketball players. Centers were significantly taller, heavier with higher fat than forwards, and heavier than guards. Higher body fat content in centers was established to increase the body frame size and facilitate physical contact (5). However, excess body fat can inhibit jumping and sprinting performance as observed by negative correlations with OFS, JFS and SFS similar as reported in other studies (9, 10, 21). Specifically, subscapular, suprailiac and triceps skinfolds presented significant predictors for OFS, JFS and SFS, respectively.

Mean overall (OFS), jumping (JFS) and sprinting (SFS) fitness scores showed that forwards had the highest athleticism, followed by centers and guards as the least athletically endowed. Anaerobic performance (jumps and sprints) results did not align with previous findings where guards had higher relative strength and power overall than centers (6, 9, 13). However, some studies (6, 45) reported that players with longer lower limbs have better vertical jump performances, and their anaerobic power is higher which supports our findings.

Present study results suggest that age was not significantly related to performance differences among playing positions and lower athleticism performance in guards is possibly attributed to immaturity, lack of playing and experience or specific selection of players. Fitness score differences do not compromise primary study results concerning the relations between playing position, morphology, and athleticism. It is important to consider the clinical relevance of even slight differences in age due to biological development as with single test performance (46), maturity has a significant similar impact on athleticism scores, respectively. Physical and physiological factors linked to both maturity and chronological age significantly impact an individual's athletic performance. Specifically, the years at peak height velocity (YAPHV) serve as a robust maturity indicator to predict basketball performance (47). Present study did not include an assessment of YAPHV, which could have provided stronger, evidence-based insights into the relationship between maturity and performance, consequently the influence on athleticism scores. Furthermore, it's essential to recognize that within the 12–16 age category, maturational differences among individuals are notably influenced by hormonal and maturational changes. These variations, often referred to as the “relative age effect,” are particularly pronounced within cohorts grouped by annual age, showcasing considerable variability. While study did not utilize maturity status, we assumed that it might impact athleticism scores but not the correlations between body measurements and performance. We acknowledge that age differences may be linked to maturity variations, and, as a result, younger players might have lower performance levels. This should be considered when interpreting differences in athleticism among playing positions (see Supplementary Table S1). As a result, the level of biological development and the age of an athlete are essential factors in the determination of athletic fitness scores. The importance of understanding and acknowledging these factors cannot be overstated.

Body height was significantly highly related to athleticism scores (distinctly OFS and JFS) in guards compared to the overall sample. Such findings support the fact that younger players are still developing in terms of longitudinal dimensions and that height is a great indicator to determine performance abilities (48). Interestingly, arm span was related to athleticism when compared to the overall sample, but not playing positions. Ziv and Lidor (49) found that arm span was positively associated with performance in several basketball skills, such as shooting and rebounding. It would be of great importance to determine whether there is a difference in the contribution of arm span among playing positions in basketball skills.

Moreover, the relationship between the amount of fat tissue and skinfolds on the arms and the effectiveness and efficiency of explosive movement would also be of great interest since the study outcomes are directing to some interesting relations. It is evident that only CMJ free arms fall within the Bland-Altman agreement of JFS (Supplementary Figures S2–S4), pointing to low triceps skinfold as a potential facilitation factor during arm swing. Fast and powerful arm movement during jumping could be inhibited by the presence of more adipose tissue in the antagonist arm region.

As previously reported lean body mass was positively associated with anaerobic power, vertical jump and sprint performance (5, 26). Similar relations for fitness scores were observed in the overall sample. However, when observed among playing positions only guards had a strong correlation between jumping performance and lean body mass as explained by Steffl et al. (50), where it was found that greater relative lean mass was associated with better performance in jump and sprint tests.

Studies (1, 5, 12, 17, 26) mainly combine different protocols to create “universal” conclusions but comparing the results between studies becomes difficult. Using a simple athleticism score approach to determine relations between performance and various specific player features can help in overcoming research that lacks longitudinal studies, inconsistencies in test protocols and sample size issues mentioned by Drinkwater et al. (26). Practical application of the overall athleticism can help in athletes' evaluation across different sport events or sports, talent identification, performance tracking, injuries assessment and to create normative data analysis.

Relying solely on fitness tests to assess overall athleticism may not capture all aspects of an individual's athletic prowess or account for sport-specific skills (8). Fitness tests typically focus on specific components of fitness, such as strength, endurance, speed, and agility. While these elements are undoubtedly important, they do not encompass the entirety of athleticism, which also includes factors like coordination, flexibility, power, and sport-specific skills. Furthermore, the assumption that the chosen fitness tests are comprehensive and equally weighted is not always warranted. Different tests may emphasize certain aspects of fitness more than others, leading to an imbalance in the assessment. In addition to the limitations of fitness tests, the sample group used for assessment should be appropriate, homogeneous, and representative.

Several study limitations were present:

- Sample size. Even though sample size was larger than the minimum calculated (n: 47 VS 41), increasing the sample size would add additional statistical power explaining effects and correlations between athleticism scores and anthropometry in basketball players more effectively. More robust athleticism scores thresholds could be gained by using a larger homogeneous sample size.
- The study provides a cross-sectional view of TSA and morphology at a specific time point within the cohort without longitudinal data. Using TSA longitudinally can provide valuable insights into trends and developmental pathways. A lack of longitudinal data impairs the ability to examine how athleticism evolves and interacts with anthropometry over time. Subsequent investigations may consider using the methodology employed in the current study across longitudinal datasets to achieve more comprehensive and conclusive outcomes.
- The study acknowledges the potential differences in maturity status between groups of playing positions but did not assess maturity of participants. Failing to account for differences in maturity status limits more persuasive conclusions by introducing confounding variables that affect the interpretation of study results. Using a simple method that involves assessing morphological parameters such as height, sitting height, leg length, and chronological age (51) to estimate maturity state could be used in future research.

Different positions require different skill sets and physical attributes seen through different levels of the obtained athleticism score. For instance, centers and power forwards often benefit from a taller stature and strength, while guards may rely more on speed, agility, and ball-handling skills. Coaches and trainers must account for these positional differences when designing training programs to ensure optimal performance. Study results suggest that playing positions have a significant impact on the body composition, anthropometry, and athleticism fitness scores of young basketball players. Given the multifaceted nature of the relationship between morphology and performance, coaches and trainers should adopt a holistic approach in developing training programs for basketball players. They should consider multiple factors, including skill level, training status, playing position, and individual goals that address the specific needs of each player and optimize their performance on the basketball court.

## 5. Conclusion

According to our knowledge, this study is the first to investigate the relationship between athleticism (as measured through fitness tests) and body composition in young basketball players. The following conclusions can be drawn:

(a) In contrast to single fitness tests, athleticism scores show a higher correlation and predictive contribution with anthropometric measures.
(b) In contrast to other studies, which have reported lower correlations between single fitness tests and anthropometric measurements, athleticism scores show a higher correlation with body composition.
(c) The variables of fat percentage, lean muscle mass, BMI, skinfold thickness, and arm span exhibited robust predictive capabilities in relation to athleticism scores.
(d) The findings revealed that forwards displayed superior athleticism in power and sprint abilities when compared to guards and centers.
(e) Even minor disparities in age and biological maturity levels within a cohort can exert a discernible influence on athleticism scores.

Furthermore, the utilization of the athleticism score holds promise in investigating disparities in body composition measurements among athletes of similar age across diverse sports, positions, or performance levels. Additionally, longitudinal studies can employ this metric to examine temporal alterations in body composition and athleticism. It is worth noting that methodological approaches, participant demographics, and assessment protocols may vary across studies, and the adoption of a universally recognized framework for assessing athleticism can help address this concern. Furthermore, it is crucial to acknowledge that athleticism is a multidimensional construct encompassing factors such as skill, coordination, speed, and agility, which collectively contribute to an individual's athleticism. Thus, while exploring the relationship between athleticism scores and body composition measures yields valuable insights, it should not serve as the sole determinant of an individual's overall athletic ability.

Coaches and practitioners should note that athleticism involves many factors beyond what has been mentioned, so assessments should consider additional factors including the player's goals, training level, experience, health, and specific needs. Different positions in basketball require different skill sets and physical traits. Coaches can use athleticism scores to design training that fits each position and needs. For instance, centers and power forwards can benefit from strength training, while guards may need agility and ball-handling drills. Also, maturity and age are important. Younger players are still developing physically, and this can affect their performance and body features-skills relation. Make sure to compare players with similar maturity levels and physical development to get a clear picture.

Additionally, coaches should:

(i) Ensure that the players sample group used for assessment is appropriate, homogeneous, and representative with no differences in maturity status.
(ii) Create and regularly update power and speed normative data for athlete assessment to establish benchmarks. This can provide context for comparing athletes' performance to their peers, ideally from the same team or with similar training regimes.
(iii) Recognize the impact of body composition on athleticism. Excess body fat can hinder performance, especially in terms of jumping and sprinting. Monitor skinfold measurements, as they can serve as predictors for certain aspects of athleticism.
(iv) Consider integrating strength and power training to improve lean body mass, especially for positions where jumping and sprinting are crucial.
(v) Consider using an overall athleticism score to assess athletes across different sports or events. This approach can help in talent identification, performance tracking, and injury assessment.

## Data Availability

The raw data supporting the conclusions of this article will be made available by the authors, without undue reservation.

## References

[1] Hoffman JR. Physiology of basketball. In: Handbook of sports medicine and science: Basketball. Hoboken, NJ: Wiley-Blackwell (2003). p. 12–24. 10.1002/9780470693896.ch2

[2] Jeličić M Ivančev V Čular D Čović N Stojanović E Scanlan AT The 30–15 intermittent fitness test: a reliable, valid, and useful tool to assess aerobic capacity in female basketball players. Res Q Exerc Sport. (2020) 91(1):83–91. 10.1080/02701367.2019.1648743 31609179

[3] Čaušević D Mašić S Doder I Matulaitis K Spicer S. Speed, agility and power potential of young basketball players. Baltic J Sport Health Sci. (2022) 4(127):29–34. 10.33607/bjshs.v127i4.1297

[4] Nikolaidis P Calleja-González J Padulo J. The effect of age on positional differences in anthropometry, body composition, physique and anaerobic power of elite basketball players. Sport Sci Health. (2014) 10(3):225–33. 10.1007/s11332-014-0198-5

[5] Ostojic SM Mazic S Dikic N. Profiling in basketball: physical and physiological characteristics of elite players. J Strength Cond Res. (2006) 20(4):740–4. 10.1519/R-15944.1 17149984

[6] Sallet P Perrier D Ferret J Vitelli V Baverel G. Physiological differences in professional basketball players as a function of playing position and level of play. J Sports Med Phys Fitness. (2005) 45(3):291–4. PMID: . 16230979

[7] Abdelkrim NB Castagna C Jabri I Battikh T El Fazaa S El Ati J. Activity profile and physiological requirements of junior elite basketball players in relation to aerobic-anaerobic fitness. J Strength Cond Res. (2010) 24(9):2330–42. 10.1519/JSC.0b013e3181e381c1 20802281

[8] Wen N Dalbo VJ Burgos B Pyne DB Scanlan AT. Power testing in basketball: current practice and future recommendations. J Strength Cond Res. (2018) 32(9):2677–91. 10.1519/JSC.0000000000002459 29401204

[9] Delextrat A Cohen D. Strength, power, speed, and agility of women basketball players according to playing position. J Strength Cond Res. (2009) 23(7):1974–81. 10.1519/JSC.0b013e3181b86a7e 19855320

[10] Nikolaidis PT Asadi A Santos EJ Calleja-González J Padulo J Chtourou H Relationship of body mass status with running and jumping performances in young basketball players. Muscles Ligaments Tendons J. (2015) 5(3):187–94. 10.11138/mltj/2015.5.3.187 26605193 PMC4617219

[11] Pehar M Sekulic D Sisic N Spasic M Uljevic O Krolo A Evaluation of different jumping tests in defining position-specific and performance-level differences in high level basketball players. Biol Sport. (2017) 34(3):263–72. 10.5114/biolsport.2017.67122 29158620 PMC5676323

[12] Delextrat A Cohen D. Physiological testing of basketball players: toward a standard evaluation of anaerobic fitness. J Strength Cond Res. (2008) 22(4):1066–72. 10.1519/JSC.0b013e3181739d9b 18545206

[13] Hoare DG. Predicting success in junior elite basketball players. The contribution of anthropometric and physiological attributes. J Sci Med Sport. (2000) 3(4):391–405. 10.1016/s1440-2440(00)80006-7 11235005

[14] Kucsa R Mačura P. Physical characteristics of female basketball players according to playing position. Acta Facultatis Educationis Physicae Universitatis Comenianae. (2015) 55(1):46–53. 10.1515/afepuc-2015-0006

[15] Rago V Pizzuto F Raiola G. Relationship between intermittent endurance capacity and match performance according to playing position in sub-19 professional male football players: preliminary results. J Phys Educ Sport. (2017) 17(2):688–91. 10.7752/jpes.2017.02103

[16] Alejandro V Santiago S Gerardo VJ Carlos MJ Vicente GT. Anthropometric characteristics of spanish professional basketball players. J Hum Kinet. (2015) 46:99–106. 10.1515/hukin-2015-0038 26240653 PMC4519226

[17] Gryko K Kopiczko A Mikolajec K Stasny P Musalek M. Anthropometric variables and somatotype of young and professional male basketball players. Sports. (2018) 6(1):9. 10.3390/sports6010009 29910313 PMC5969204

[18] Dežman B Trninic S Dizdar D. Expert model of decision-making system for efficient orientation of basketball players to positions and roles in the game–empirical verification. Coll Antropol. (2001) 25(1):141–52. PMID: . 11787538

[19] Gaetano A Tiziana DI Di Tore PA. Anthropometrics characteristics and jumping ability in basketball. J Hum Sport Exerc. (2018) 13:385–92. 10.14198/jhse.2018.13.Proc2.22

[20] Nunes JA Aoki MS Altimari LR Petroski EL Júnior DDR Montagner PC. Anthropometric profile and indicators of playing performance in Brazilian women s Olympic basketball teams. Revista Brasileira de Cineantropometria e Desempenho Humano. (2009) 11(1):67–72. 10.5007/1980-0037.2009v11n1p67

[21] Ribeiro BG Mota HR Sampaio-Jorge F Morales AP Leite TC. Correlation between body composition and the performance of vertical jumps in basketball players. J Exerc Physiol Online. (2015) 18(5):69–79. https://www.asep.org/asep/asep/JEPonlineOCTOBER2015_Morales.pdf

[22] Kalinski M Norkowski H Kerner M Tkaczuk W. Anaerobic power characteristics of elite athletes in national level team-sport games. Eur J Sport Sci. (2002) 2(3):1–21. 10.1080/17461390200072303

[23] Lohman TG Caballero B Himes JH Hunsberger S Reid R Stewart D Body composition assessment in American Indian children. Am J Clin Nutr. (1999) 69(4):764S–6S. 10.1093/ajcn/69.4.764S 10195600

[24] Wilson RS David GK Murphy SC Angilletta MJ Jr Niehaus AC Hunter AH Skill not athleticism predicts individual variation in match performance of soccer players. Proc R Soc B Biol Sci. (2017) 284(1868):20170953. 10.1098/rspb.2017.0953 PMC5740267 29187623

[25] Turner AN. Total score of athleticism: a strategy for assessing an athlete’s athleticism. Prof Strength Cond. (2014) 33:13–7. 10.1519/SSC.0000000000000506

[26] Drinkwater EJ Pyne DB McKenna MJ. Design and interpretation of anthropometric and fitness testing of basketball players. Sports Med. (2008) 38(7):565–78. 10.2165/00007256-200838070-00004 18557659

[27] Philipp NM Crawford DA Fry AC. A total score of athleticism to estimate the amount of variance explained in on-field performance within collegiate American football players. Int J Strength Cond. (2022) 2(1):1–9. 10.47206/ijsc.v2i1.94

[28] Turner AN Jones B Stewart P Bishop C Parmar N Chavda S Total score of athleticism: holistic athlete profiling to enhance decision-making. Strength Cond J. (2019) 41(6):91–101. 10.1519/SSC.0000000000000506

[29] Till K Jones BL Cobley S Morley D O'Hara J Chapman C Identifying talent in youth sport: a novel methodology using higher-dimensional analysis. PloS One. (2016) 11(5):e0155047. 10.1371/journal.pone.0155047 27224653 PMC4880304

[30] Maestroni L Turner A Papadopoulos K Sideris V Read P. Total score of athleticism: profiling strength and power characteristics in professional soccer players after anterior cruciate ligament reconstruction to assess readiness to return to sport. Am J Sports Med. (2023). 10.1177/03635465231194778 37681510 PMC10543956

[31] Oleksy Ł Mika A Królikowska A Kuchciak M Stolarczyk M Kielnar R Composite score of readiness (csr) as holistic profiling of functional deficits in footballers following ACL reconstruction. J Clin Med. (2021) 10(16):3570. 10.3390/jcm10163570 34441865 PMC8397164

[32] Bourdon PC Cardinale M Murray A Gastin P Kellmann M Varley MC Monitoring athlete training loads: consensus statement. Int J Sports Physiol Perform. (2017) 12(s2):S2-161–70. 10.1123/IJSPP.2017-0208 28463642

[33] Faul F Erdfelder E Lang A-G Buchner A. G* power 3: a flexible statistical power analysis program for the social, behavioral, and biomedical sciences. Behav Res Methods. (2007) 39(2):175–91. 10.3758/BF03193146 17695343

[34] Cossio-Bolaños M Vidal-Espinoza R de Campos LFCC Sulla-Torres J Cossio-Bolaños W Albornoz CU Equations predicting maturity status: validation in a cross-sectional sample to assess physical growth and body adiposity in Chilean children and adolescents. Endocrinología, Diabetes y Nutrición (Engl Ed.). (2021) 68(10):689–98. 10.1016/j.endien.2021.11.033 34924157

[35] Čaušević D Čović N Abazović E Rani B Manolache GM Ciocan CV Predictors of speed and agility in youth male basketball players. Appl Sci. (2023) 13:7796. 10.3390/app13137796

[36] Arslan E Kilit B Clemente FM Murawska-Ciałowicz E Soylu Y Sogut M Effects of small-sided games training versus high-intensity interval training approaches in young basketball players. Int J Environ Res Public Health. (2022) 19(5):2931. 10.3390/ijerph19052931 35270619 PMC8910324

[37] Zhang M Liang X Huang W Ding S Li G Zhang W The effects of velocity-based versus percentage-based resistance training on athletic performances in sport-collegiate female basketball players. Front Physiol. (2023) 10(13):992655. 10.3389/fphys.2022.992655 PMC9873382 36703922

[38] Dalleau G Belli A Viale F Lacour JR Bourdin M. A simple method for field measurements of leg stiffness in hopping. Int J Sports Med. (2004) 25(03):170–6. 10.1055/s-2003-45252 15088239

[39] Mukaka MM. A guide to appropriate use of correlation coefficient in medical research. Malawi Med J. (2012) 24(3):69–71. PMCID: . 23638278 PMC3576830

[40] Hopkins W. A spreadsheet for combining outcomes from several subject groups. Sportscience. (2006) 10:51–3.

[41] Carson C. The effective use of effect size indices in institutional research. 31st Annual conference proceedings (vol. 41) (2012).

[42] Jeličić M Sekulić D Marinović M. Anthropometric characteristics of high level European junior basketball players. Coll Antropol. (2002) 26(2):69–77. PMID: . 12674837

[43] Vigo A Viviani F. The adolescent basketball player: the importance of some anthropometric characteristics for speed, resistance, power and agility. Antrocom. (2020) 16:2.

[44] Boone J Bourgois J. Morphological and physiological profile of elite basketball players in Belgium. Int J Sports Physiol Perform. (2013) 8(6):630–8. 10.1123/ijspp.8.6.630 23475191

[45] Aouadi R Jlid MC Khalifa R Hermassi S Chelly MS Van Den Tillaar R Association of anthropometric qualities with vertical jump performance in elite male volleyball players. J Sports Med Phys Fitness. (2012) 52(1):11–7. PMID: . 22327081

[46] Čaušević D Rani B Gasibat Q Čović N Alexe CI Pavel SI Maturity-Related variations in morphology, body composition, and somatotype features among young male football players. Children. (2023) 10(4):721. 10.3390/children10040721 37189970 PMC10136423

[47] Torres-Unda J Zarrazquin I Gravina L Zubero J Seco J Gil SM Basketball performance is related to maturity and relative age in elite adolescent players. J Strength Cond Res. (2016) 30(5):1325–32. 10.1519/JSC.0000000000001224 26439783

[48] Hernández S Ramirez-Campillo R Álvarez C Sanchez-Sanchez J Moran J Pereira LA Effects of plyometric training on neuromuscular performance in youth basketball players: a pilot study on the influence of drill randomization. J Sports Sci Med. (2018) 17(3):372–8. PMCID: . 30116110 PMC6090388

[49] Ziv G Lidor R. Physical attributes, physiological characteristics, on-court performances and nutritional strategies of female and male basketball players. Sports Med. (2009) 39:547–68. 10.2165/00007256-200939070-00003 19530751

[50] Steffl M Bohannon RW Sontakova L Tufano JJ Shiells K Holmerova I. Relationship between sarcopenia and physical activity in older people: a systematic review and meta-analysis. Clin Interv Aging. (2017) 12:835–45. 10.2147/CIA.S132940 28553092 PMC5441519

[51] Mirwald RL Baxter-Jones AD Bailey DA Beunen GP. An assessment of maturity from anthropometric measurements. Med Sci Sports Exerc. (2002) 34(4):689–94. 10.1097/00005768-200204000-00020 11932580

