# Supplementary material for: Relations between specific athleticism and morphology in young basketball players
Source: Front Sports Act Living. 2023 Oct 9;5:1276953. doi: 10.3389/fspor.2023.1276953 (PMC10593457; doi:10.3389/fspor.2023.1276953)

**Figure S1.** Differences in Effect size (ES) between playing positions for Overall Fitness Score (OFS), Jumping Fitness Score (JFS) and Sprinting Fitness Score (SFS) in young basketball players.


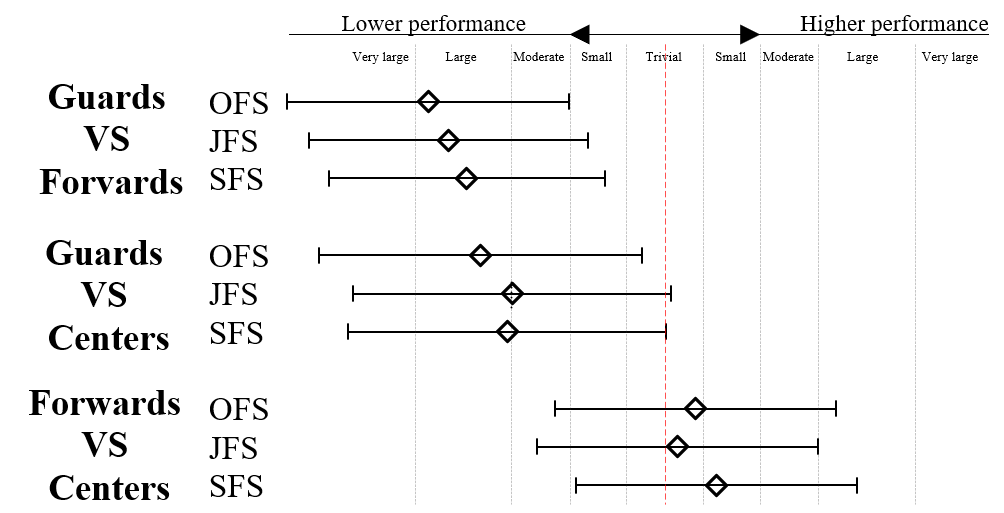


**Table S1.** Limits of decision for the difference based on Pearson’s r correlations between playing positions for Overall Fitness Score (OFS), Jumping Fitness Score (JFS) and Sprinting Fitness Score (SFS).

|  | Overall Fitness Score | | |  | Jumping Fitness Score | | |  | Sprinting Fitness Score | | |
| --- | --- | --- | --- | --- | --- | --- | --- | --- | --- | --- | --- |
|  | Guards  vs  Forwards | Guards  vs  Centers | Forwards  vs  Centers |  | Guards  vs  Forwards | Guards  vs  Centers | Forwards  vs  Centers |  | Guards  vs  Forwards | Guards  vs  Centers | Forwards  vs  Centers |
| Body height | ++ | ? | - |  | + + | ? | - |  | ? | ? | ? |
| Body mass | ? | ? | ? |  | ? | ? | ? |  | ? | ? | ? |
| BMI | ? | ? | ? |  | ? | ? | ? |  | ? | ? | ? |
| Fat free mass | ? | ? | ? |  | + | ? | ? |  | ? | ? | ? |
| Fat % | ? | ? | ? |  | ? | ? | ? |  | ? | ? | ? |
| Lean body mass | ? | ? | ? |  | ? | ? | ? |  | ? | ? | ? |
| Standing reach | + | ? | - |  | + | ? | - - |  | ? | ? | ? |
| Arm span | ? | ? | ? |  | + | ? | - |  | ? | ? | ? |
| Hand span | ? | ? | - |  | ? | ? | ? |  | ? | - | - |
| Suprailiac skinfold | ? | ? | ? |  | ? | + | ? |  | ? | ? | + |
| Subscapular skinfold | ? | + | + |  | ? | + | ? |  | ? | ? | + |
| Biceps skinfold | ? | ? | ? |  | ? | ? | ? |  | ? | ? | ? |
| Triceps skinfold | ? | ? | ? |  | ? | ? | ? |  | ? | ? | ? |
| *++ Very likely positive; + Likely positive; - - Very likely negative; - Likely negative; ?- Unclear* | | | | | | | | | | | |

**Figure S2.** Bland-Altman plot for the limits of agreement between: a) Overall Fitness Score (OFS) and Jumping Fitness Score (JFS), b) Overall Fitness Score (OFS) and Sprinting Fitness Score SFS and c) Jumping Fitness Score (JFS) and Sprinting Fitness Score SFS.


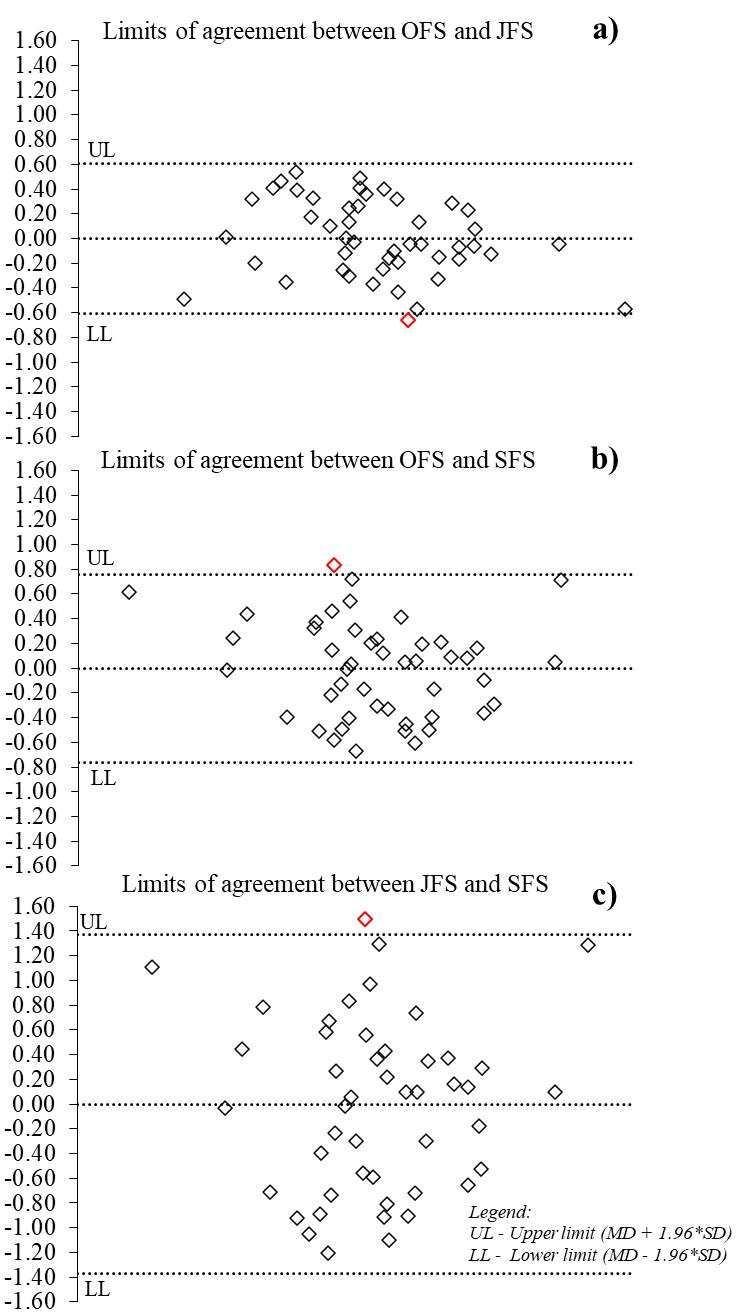


**Figure S3.** Bland-Altman plot for the limits of agreement between Jumping Fitness Score (JFS) and a) Countermovement jump (CMJ), b) Countermovement free arms (CMJ free arms), c) Drop jump from 40 cm (DJ 40cm), d) Stiffness test with 10 jumps (STIFNESS 10 jumps) and e) standing long jump as comprehensive measure of explosive power.


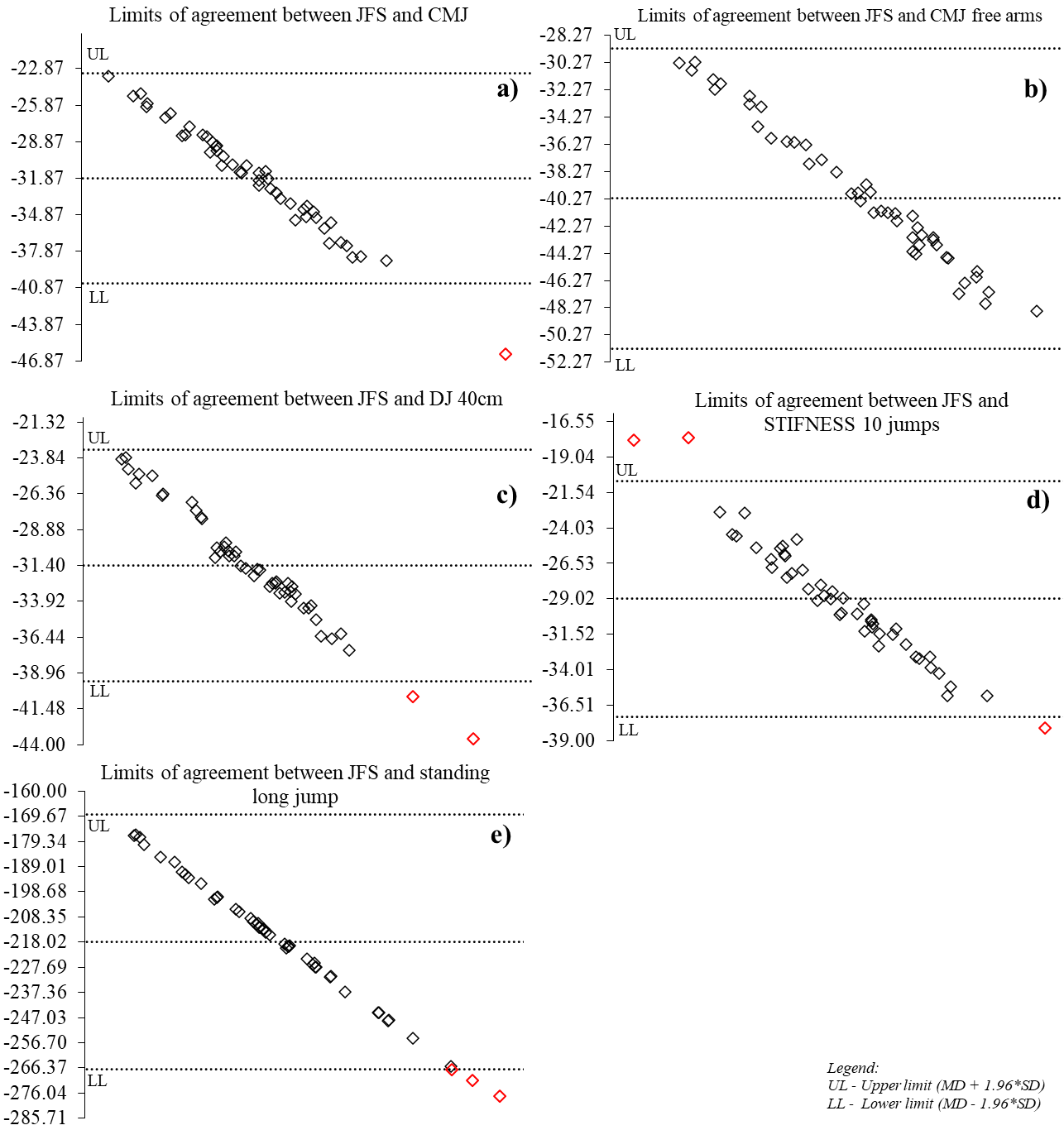


**Figure S4.** Bland-Altman plot for the limits of agreement between Sprinting Fitness Score (SFS) and a) sprint time on 5 meters (5m sprint), b) sprint time on 10 meters (10m sprint), c) sprint time on 15 meters (15m sprint) and d) sprint time on 20 meters (20m sprint) as comprehensive measure of speed.


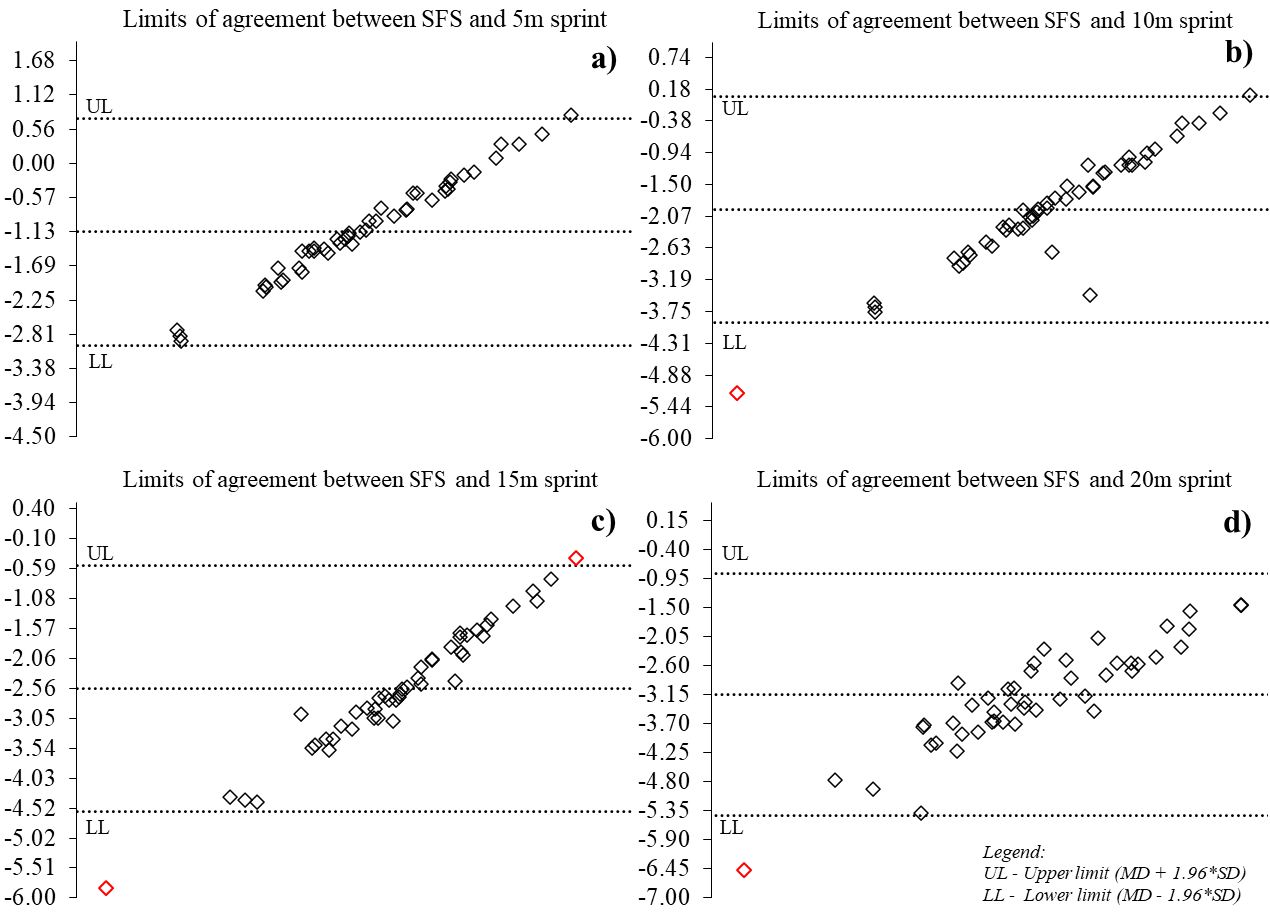

Supplement: Supplementary file 1 [file Datasheet1.docx]
